# Nontyphoidal *Salmonella* Bacteremia Resulting in Thoracic Aortic Dissection

**DOI:** 10.4269/ajtmh.16-0355

**Published:** 2016-11-02

**Authors:** Simon Smith, Queen Okereke, Josh Hanson

**Affiliations:** 1 Department of Medicine, Cairns Hospital, Cairns, Australia; 2 James Cook University, Cairns, Australia; 3 Menzies School of Health Research, Darwin, Australia

**Figure 1.**
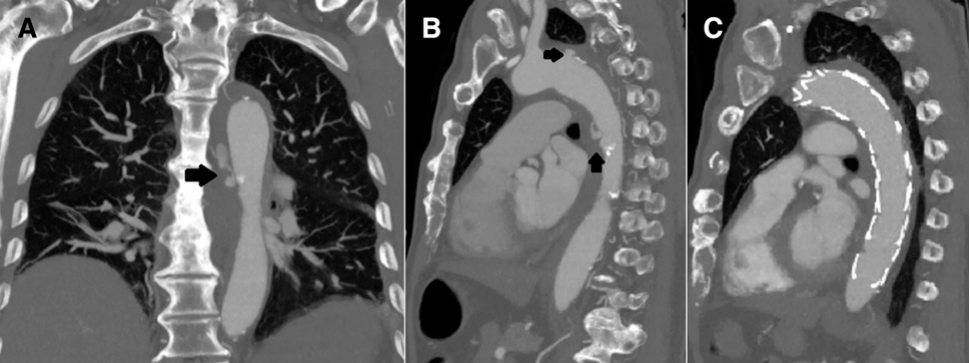
(**A**) Coronal images revealing extravasation of contrast at the site of the calcified atherosclerotic plaque (arrow). (**B**) Sagittal images highlighting the calcified plaque with adjacent leak of contrast from the proximal (upper arrow) and mid-descending (lower arrow) thoracic aorta. (**C**) Sagittal image showing repair of the aortic dissection with graft in situ.

An 84-year-old male retired boilermaker, residing in Australia, presented with the acute onset of anterior, left-sided, pleuritic chest pain. He had a history of ischemic heart disease, and had been on a long-haul flight, returning from Mauritius 9 days previously. An electrocardiogram and echocardiogram were normal and serum troponin levels were negative. A computed tomography (CT) pulmonary angiogram excluded pulmonary embolism, and revealed a calcified atherosclerotic plaque in the thoracic aorta but no other abnormalities. He had no history of diarrhea. On day 2 of admission, he became pyrexial with ongoing chest pain. Blood cultures grew *Salmonella typhimurium* on three separate collections and intravenous ceftriaxone was initiated. To investigate his ongoing chest pain, on day 7, a CT aortogram was performed, which demonstrated contrast leaking at the site of the calcified atherosclerotic plaque in the proximal and mid-descending thoracic aorta (Figure 1

). The aorta was repaired endovascularly by placement of two woven polyester thoracic grafts; the first to the distal aorta above the celiac artery and the second 5 mm distal to the left subclavian artery. Repeat imaging revealed no evidence of ongoing leak. Post-procedure blood cultures were negative. The patient received 4 weeks of intravenous ceftriaxone, and was prescribed oral ciprofloxacin for life-time suppression. Six weeks postoperatively, a repeat CT aortogram revealed no leak, his anemia was improving, and his C-reactive protein had normalized.

Endovascular infection caused by nontyphoidal *Salmonella* is well described; however, it usually involves the abdominal aorta.^1^ Patients over the age of 50 years with risk factors for atherosclerotic disease are most likely to develop aortitis secondary to *Salmonella* bacteremia.^2^,^3^ Medical therapy alone is unlikely to be curative; hence, patients should also receive surgical intervention in conjunction with extended antibiotic therapy.^4^

Written informed consent was obtained from the patient and provided to the Editor-In-Chief of this journal.
